# Using case vignettes to study the presence of outcome, hindsight, and implicit bias in acute unplanned medical care: a cross-sectional study

**DOI:** 10.1097/MEJ.0000000000001127

**Published:** 2024-02-16

**Authors:** Patricia Plaum, Laura N. Visser, Bas de Groot, Marlies E.B. Morsink, Wilma L.J.M. Duijst, Bart G.J. Candel

**Affiliations:** a Emergency Department, Zuyderland Medical Centre, Heerlen; b Emergency Department, Máxima Medical Centre, Veldhoven; c Emergency Department, Radboud University Medical Centre, Nijmegen; d Faculty of Law and Criminology, Maastricht University, Maastricht; e GGD IJsselland, Zwolle; f Emergency Department, Leiden University Medical Centre, Leiden, The Netherlands; g Emergency Department, Fiona Stanley Hospital, Perth, Australia

**Keywords:** bias, chronic fatigue syndrome, disciplinary law, fibromyalgia, functional disorders, health care, irritable bowel syndrome, judgment, medical errors, outcome assessment

## Abstract

**Background and importance:**

Various biases can impact decision-making and judgment of case quality in the Emergency Department (ED). Outcome and hindsight bias can lead to wrong retrospective judgment of care quality, and implicit bias can result in unjust treatment differences in the ED based on irrelevant patient characteristics.

**Objectives:**

First, to evaluate the extent to which knowledge of an outcome influences physicians’ quality of care assessment. Secondly, to examine whether patients with functional disorders receive different treatment compared to patients with a somatic past medical history.

**Design:**

A web-based cross-sectional study in which physicians received case vignettes with a case description and care provided. Physicians were informed about vignette outcomes in a randomized way (no, good, or bad outcome). Physicians rated quality of care for four case vignettes with different outcomes. Subsequently, they received two more case vignettes. Physicians were informed about the past medical history of the patient in a randomized way (somatic or functional). Physicians made treatment and diagnostic decisions for both cases.

**Setting and participants:**

One hundred ninety-one Dutch emergency physicians (EPs) and general practitioners (GPs) participated.

**Outcome measures and analysis:**

Quality of care was rated on a Likert scale (0–5) and dichotomized as adequate (yes/no). Physicians estimated the likelihood of patients experiencing a bad outcome for hindsight bias. For the second objective, physicians decided on prescribing analgesics and additional diagnostic tests.

**Main results:**

Large differences existed in rated quality of care for three out of four vignettes based on different case outcomes. For example, physicians rated the quality of care as adequate in 44% (95% CI 33–57%) for an abdominal pain case with a bad outcome, compared to 88% (95% CI 78–94%) for a good outcome, and 84% (95% CI 73–91%) for no outcome (*P* < 0.01). The estimated likelihood of a bad outcome was higher if physicians received a vignette with a bad patient outcome. Fewer diagnostic tests were performed and fewer opioids were prescribed for patients with a functional disorder.

**Conclusion:**

Outcome, hindsight, and implicit bias significantly influence decision-making and care quality assessment by Dutch EPs and GPs.

## Introduction

Emergency medicine has a high density of crucial decisions in an increasingly complex acute healthcare system, which may lead to errors [1,2]. To process these decisions, physicians tend to rely on heuristic strategies (decisional shortcuts), based on recognized patterns, experience and habit [3]. However, these strategies introduce cognitive biases, potentially compromising patient care and perceived quality. While there are various cognitive biases [4], our study focuses on three prevalent in unplanned medical care: outcome, hindsight and implicit bias. These biases are part of daily decision-making, impacting different physicians and patient groups.

Two important aspects of unplanned medical care are to learn from individual cases to improve patient care and evaluate its quality. Therefore, many cases are judged retrospectively by physicians in morbidity and mortality meetings. However, retrospective judgment may be prone to several forms of bias [5,6]. Outcome bias is the impact of outcome knowledge on evaluations of decision quality. Hindsight bias refers to the exaggerated extent to which individuals indicate they would have predicted the event beforehand, given knowledge of an outcome. Both outcome and hindsight bias may negatively impact retrospective investigations of an erroneous event, leading to wrong judgment of the quality of care and clinical performance of physicians, sometimes leading to burn-out [7].

Implicit bias, another significant factor in unplanned medical care, involves unconscious negative evaluations of one group relative to another. For instance, racial implicit bias influences treatment decisions, affecting prescription practices in healthcare [8–10]. Vulnerable groups, such as patients with functional disorders [e.g. irritable bowel syndrome (IBS), chronic fatigue syndrome (CFS), fibromyalgia], often feel their concerns are dismissed, and implicit bias may exacerbate disparities in care [11,12]. Sex differences, particularly in conditions more prevalent in females, further complicate care delivery [13].

This study aims to explore the influence of outcome knowledge on physician assessments and investigate how sex and medical history impact clinical decision-making in emergency care. Notably, the study raises awareness about the potential pitfalls of extrapolating findings from a previous study from the USA to the European context, emphasizing the need for caution and scrutiny due to cultural, institutional, and legal differences [5,14–16]. Ultimately, the goal is to enhance understanding and address biases to improve the quality and equality of care in emergency medicine.

## Methods

### Study design and setting

This web-based cross-sectional survey was performed in the Netherlands between 11 August 2022 and 14 May 2023. Reporting adheres to the STROBE guidelines. Emergency physicians (EPs) and general practitioners (GPs) were included as they both experience undifferentiated and acute unplanned medical care. Both EPs and GPs have a 3-year training program in the Netherlands. Most EPs and GPs in training (residents), already have junior resident experience. The study was approved by the medical ethics committee of Máxima Medical Center (N22.025) and registered on clinicaltrials.gov (NCT05424497).

### Participants

All 767 EPs in the Netherlands, including EPs in training, were invited by email through the Dutch Association of Emergency Physicians (NVSHA) to participate in this study. Approximately 100 GPs (including GPs in training) also received information about the study by word of mouth, and emails to certain GP practices throughout the Netherlands. Informed consent was obtained digitally before inclusion.

### Study procedure

Participants received six vignettes by email. Four were used to study outcome and hindsight bias, and two were used to study implicit bias. In the invitation, participants were asked to complete the questionnaire in a quiet environment, which would approximately take 20-30 min. The study invitation did not contain any information about the exact research purpose but did describe that the aim was to investigate how participants would evaluate certain clinical cases that were common in daily practise. The vignettes and questions were presented through an online survey using Research Manager (ResearchManager, Deventer, The Netherlands). For the first objective, four cases were used in a previous American study with a similar objective. Not all six cases were used as this would make the study significantly longer, which could lead to a lower response rate. Selection of cases was based on common presentations and varying quality of care ranging from outstanding to poor quality (headache/excellent quality, abdominal pain/good quality, trauma/below average quality, and chest pain/poor quality) [5]. Before use, the vignettes were translated and modified (e.g. medication, units) based on Dutch guidelines by two Dutch EPs working in the UK and Australia (see Supplemental digital content 1, http://links.lww.com/EJEM/A427). Participants were randomized by ResearchManager, and could get a clinical scenario with a positive, a negative or no outcome. For example, a good or positive outcome was ‘the patient did well the next day’, versus a negative or bad outcome ‘the patient developed a pulmonary embolism’. Participants received the four cases in different sequences with the aim of analyzing whether the randomization sequence affected judgment (see Supplemental digital content 2, http://links.lww.com/EJEM/A427). For the second objective, two cases written by the investigators were presented (see Supplemental digital content 1, http://links.lww.com/EJEM/A427). Participants were randomized in four groups, in which sex and the past medical history (functional or somatic/no past medical history) of the patient in the case differed (see Supplemental digital content 2, http://links.lww.com/EJEM/A427). Participants were asked if they would prescribe analgesics (yes/no), and specifically opioids (yes/no), and how many opioids daily (8 options between 0 and 60 mg). For the first (abdominal pain) case, participants were also asked whether they would perform an ultrasound to rule out an appendicitis. For the second (chest pain) case, participants were asked whether they would perform an electrocardiogram, test for troponins, and give a trial of nitro-glyceryl trinitrate sublingual (yes/no). At the end of the study, several more questions were asked to study whether explicit bias played a role in clinical judgment, which refers to biased attitude we are aware of, contrasting to implicit bias which we are unaware of. The additional questions were whether physicians thought that men or women and patients with a past medical history of fibromyalgia, depression, and CFS or no past medical history have an equal risk (similar/lower/higher) to have an appendicitis or an acute coronary syndrome.

### Data collection

The following participant characteristics were collected: age, sex, specialization (GP/EP), in training, and years of clinical experience, and experience as expert with a disciplinary law committee.

### Outcome measures

For the first objective, the primary outcome was the quality of care rated on a six-point Likert scale from 0 to 5: poor, below average, average, good, very good and outstanding. Additionally, as secondary outcome, we investigated the quality of care as a dichotomous outcome (adequate/not adequate). To study hindsight bias, participants estimated the probability of a bad outcome (in percent) for each vignette.

For the second objective, the primary outcome was whether pain medication was prescribed (yes/no) and if diagnostic investigations were performed (yes/no) per case. Secondary outcomes were whether opioids were prescribed, the dosage of opioids prescribed and which diagnostics they would have performed.

### Sample size calculation

The minimal sample size was calculated at 162 participants (see Supplemental digital content 3, http://links.lww.com/EJEM/A427).

### Data analyses

Participant characteristics were described as mean (SD) for normally distributed data, and median (interquartile range) for skewed data. Categorical data were presented as number (N (%)). For objective 1, answers from participants on the vignettes to study outcome bias (Likert scale 0–5) and hindsight bias were compared among cases with a good, bad or no outcome, using a Kruskal–Wallis test. A Chi-square test was used to compare whether the quality of care was rated as adequate or not. Additionally, to evaluate whether other variables affected the outcome (e.g. the rated quality of care) a multivariable logistic regression by forced entry of variables was performed with the following variables: age, years of experience, sex, specialism (GP, EP or EP in training, other) randomization sequence for the received vignettes and which outcome participants received (no, good, or bad outcome).

For objective 2, chi-square tests were used to compare outcomes. To investigate whether explicit bias was present instead of implicit bias, multivariable logistic regressions were performed with the variables: age, years of experience, sex, specialism, sex of patient, past medical history of patient, answer of questions for explicit bias. For the regression analysis of case 5, we also added the case outcome of case 2 from objective 1, as the bad outcome was a perforated appendicitis, which might have influenced the clinical decisions in the abdominal pain case. Similarly, we added outcome of case 4 to the regression analysis of case 6, as they were both about chest pain. Outcomes were reported with 95%-confidence intervals and *P* < 0.05 was considered significant. Data were stored in an encrypted account in ResearchManager which could only be entered by the researchers. Data analyses were performed with IBM SPSS Statistics (version 29).

## Results

In total, 191 participants were included (see Supplemental digital content 4, http://links.lww.com/EJEM/A427 for a flow diagram). Participant characteristics are described in Table 1. Most included participants were EPs (58%).

**Table 1:** Participant characteristics

Total number of participants	191
Age, mean (SD)	39.9 (9.1)
Female, n (%)	118 (55.9)
Years of clinical experience, mean (SD)	13.7 (8.5)
0–3 years, n (%)	16 (8.4)
4–6 years, n (%)	25 (13.1)
7–9 years, n (%)	21 (11.0)
10+ years, n (%)	129 (67.5)
Experience in Medical Disciplinary Law Committee	1 (0.5%)
Specialism	
Emergency physician, n (%)	122 (57.8)
Emergency physician in training, n (%)	27 (12.8)
General practitioner, n (%)	24 (12.8)
General practitioner in training and other^a^, n (%)	18 (8.5)

a Other positions can include retired General practitioners or retired Emergency physicians working in a different field.

### Outcome and hindsight bias

**Fig. 1:**
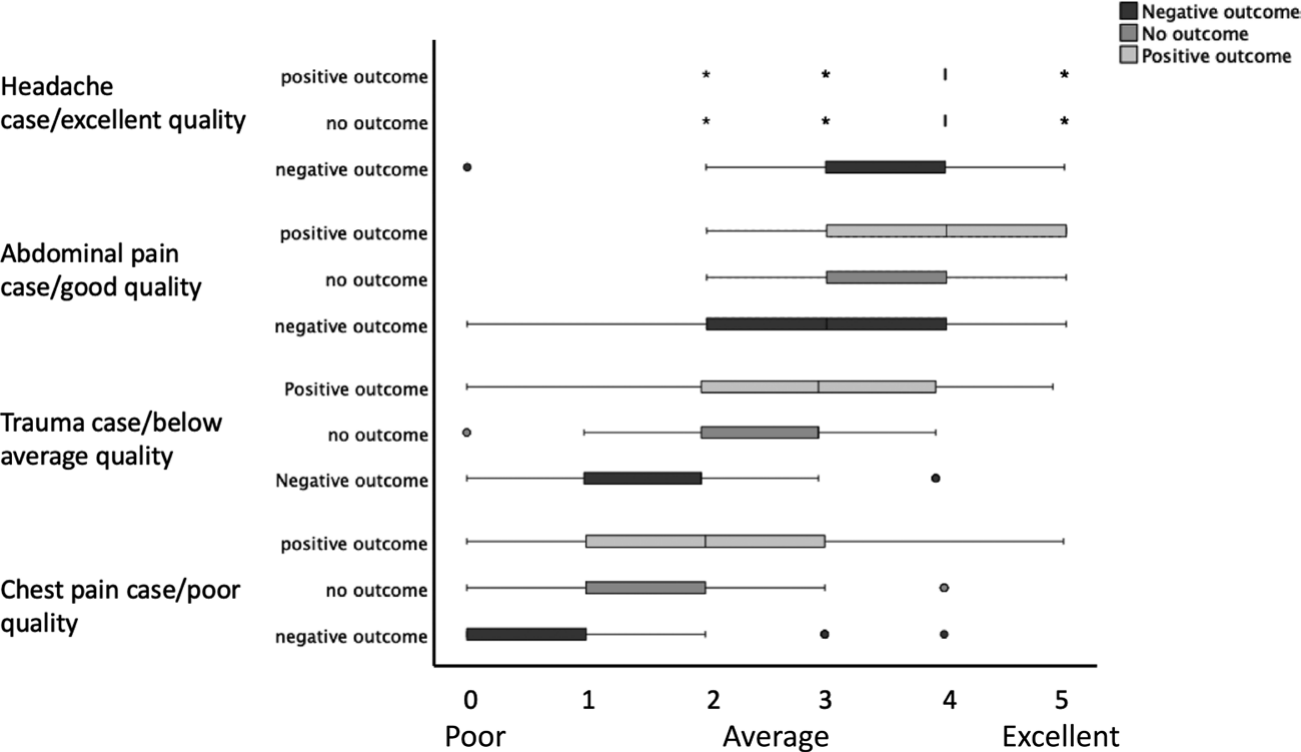
The rated quality of care on a Likert scale. The box plot shows the rated quality of care on a Likert scale (0–5) per case and per outcome. No difference in the quality of care existed among groups for the headache case (*P* = 0.80). However, for the other three cases, varying from poor to good predefined quality of care, the Likert scale was significantly different (*P* < 0.01) with lower ratings for a bad (negative) outcome compared to a good (positive) or no outcome. For the headache case, the median and IQR were all at a rating of 4 for a good and no outcome.

**Fig. 2:**
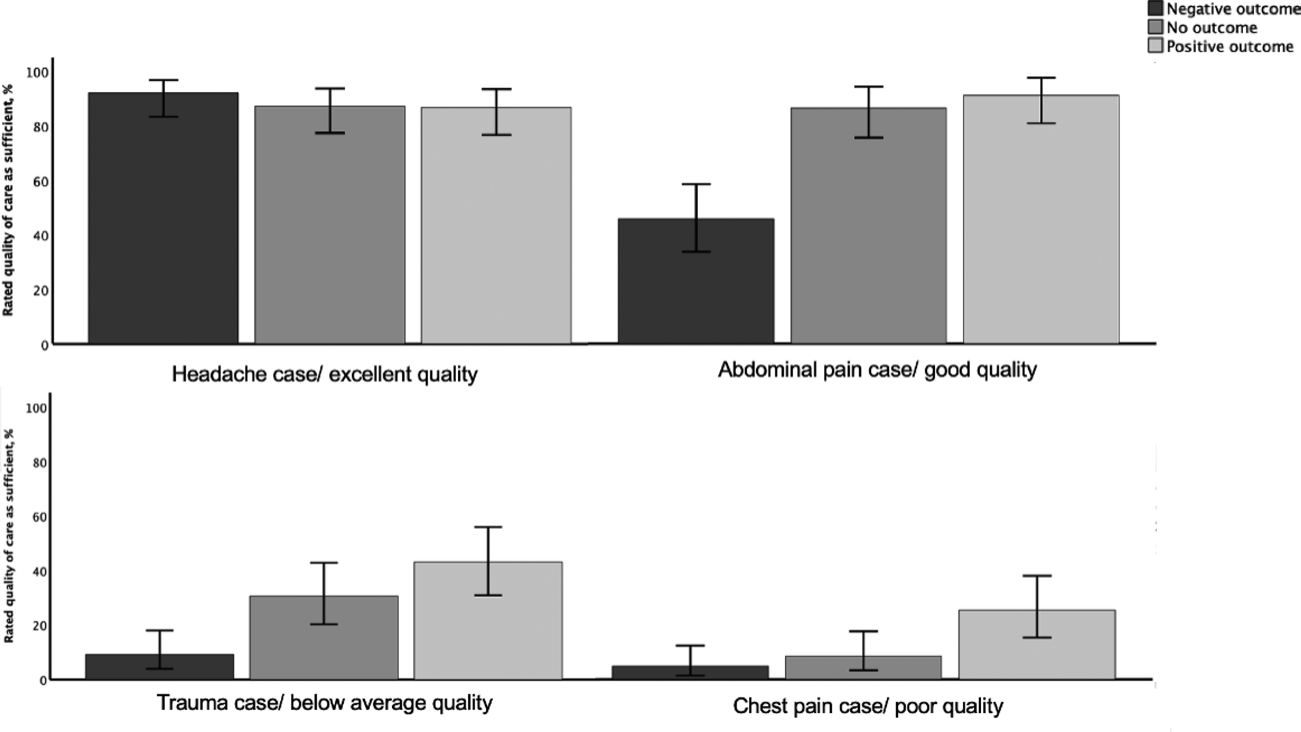
The rated quality of care as sufficient. This figure shows the percentage of cases rated as ‘adequate quality of care’ per outcome for each case. There was no difference in the rated quality of care for the headache case among the different outcomes (*P* = 0.60). However, for the other cases, the care in the vignettes with a bad (negative) outcome were significantly less often rated as adequate (*P* < 0.01) compared to no outcome or a good (positive) outcome: for the abdominal pain case 44% (95% CI 33–57%) vs 84% (95% CI 73–91) vs 88% (95% CI 78–94%), respectively, for the trauma case 9% (95% CI 4–19%) vs 31% (95% CI 21–43%) vs 43% (95% CI 31–56%), respectively, and for the chest pain case 5% (95% CI 2–13%) vs 8% (95% CI 4–18%) vs 24% (95% CI 15–37%), respectively.

For objective 1, rated quality of care was similar for all different case outcomes in the headache case (excellent predefined quality of care, *P* = 0.80). However, for the other three cases, in quality of care was rated significantly lower (*P* < 0.01) for a negative outcome compared to a positive or no outcome (Fig. 1). Figure 2 shows the percentage of cases rated as ‘adequate quality of care’ per case outcome for each case. Again, rated quality of care for the headache case among different case outcomes was similar (*P* = 0.60), but quality of care was rated more often as inadequate for the other cases if physicians received a vignette with a bad outcome (*P* < 0.01). Other variables did not affect the rated quality of care (see table in Supplemental digital content 5, http://links.lww.com/EJEM/A427). The estimated likelihood of a bad outcome was higher if participants received a negative outcome, compared to a positive or no outcome (Table 2), except for the headache case.

**Table 2:** Hindsight bias

Vignette	Outcome	Likelihood of a bad outcome (95% CI)	*P*-value
Headache case	Good outcome	7.4% (4.8–9.9)	0.48
	No outcome	6.6% (4.2–8.9)	
	Bad outcome	8.0% (4.8–11.3)	
Abdominal pain case	Good outcome	20.0% (15.5–24.4)	<0.01
	No outcome	14.9% (10.9–18.9)	
	Bad outcome	32.4% (26.7–38.1)	
Trauma case	Good outcome	31.5% (24.9–38.1)	0.02
	No outcome	32.1% (25.9–38.4)	
	Bad outcome	43.5% (36.4–50.2)	
Chest pain case	Good outcome	38.2% (31.4–45.1)	<0.01
	No outcome	49.2% (41.8–56.7)	
	Bad outcome	62.2% (56.4–67.9)	

Kruskal–Wallis tests were used to test for statistical significance.

### Implicit bias

For the abdominal pain case (objective 2), an ultrasound was less often performed for men than for women and less often for patients with a functional disorder than patients with a somatic past medical history (Table 3). For the chest pain case, opioids were less often prescribed for patients with a functional disorder, and the total dosage of opioids was lower. Adjusted for other variables, including answers to test for explicit bias, these differences persisted (see table in Supplemental digital content 6, http://links.lww.com/EJEM/A427). Troponins were more often performed for patients with a somatic past medical history than for patient with a functional disorder, which was statistically significant after adjusting for other variables.

**Table 3:** Pain relief and investigations for cases with different sex and medical history

Abdominal pain case	Female N = 92	Male N = 93	*P*-value	Functional N = 93	Somatic N = 92	*P*-value
Pain relief prescribed N (%)	87 (95%)	88 (95%)	0.99	86 (93%)	89 (97%)	0.20
Opioid prescribed N (%)	45 (49%)	41 (44%)	0.51	37 (40%)	49 (53%)	0.07
Ultrasound performed N (%)	83 (90%)	73 (79%)	0.03	70 (75%)	86 (94%)	<0.01

Chest pain case	Female N = 91	Male N = 92		Functional N = 92	Somatic N = 91	
Pain relief prescribed N (%)	69 (76%)	65 (71%)	0.43	66 (72%)	68 (75%)	0.65
Opioid prescribed N (%)	52 (57%)	47 (51%)	0.41	43 (47%)	56 (62%)	0.05
Opioid quantity N (%) 0 mg 0–20 mg 20–40 mg 40-60 mg	39 (43%) 43 (47%) 9 (9.9%) 0 (0.0%)	42 (47%) 35 (39%) 11 (12%) 2 (2.2%)	0.37	49 (54%) 34 (38%) 6 (6.7%) 1 (1.1%)	32 (35%) 44 (48%) 14 (15%) 1 (1.1%)	0.05
GTN spray N (%)	7 (7.7%)	11 (12%)	0.33	11 (12%)	7 (7.7%)	0.33
ECG N (%)	89 (98%)	89 (97%)	0.66	89 (97%)	89 (98%)	0.66
Troponins N (%)	82 (90%)	74 (80%)	0.07	74 (80%)	82 (90%)	0.07

Chi-squared tests were used to test for significant differences between the groups.

ECG, electrocardiogram; GTN, glyceryl trinitrate; N, number.

## Discussion

The present study has two main findings: First, retrospective judgment of the quality of care of cases by EPs and GPs is affected by outcome and hindsight bias. Second, clinical decision-making is affected by implicit bias, which may cause care disparities for patients with a functional disorder.

The results of objective 1 are in line with previous studies studying outcome bias in emergency medicine [5,6], and other fields [17–19]. One of our aims was to evaluate whether the results of the study from Gupta *et al*. could be extrapolated to a different culture in Europe [5,14–16]. Our study showed similar results, with non-significant differences in judgment for the headache case and large differences for the other cases. As Gupta *et al*. suggested, outcome bias may be more present in cases with a lower quality of care than with excellent care. The bad outcome for vignette 1 is rare and all physicians recognized this independent of the case outcome they received, possibly explaining the absence of outcome bias. For the other three cases, the estimated likelihood for a bad outcome, and the percentage of participants who rated quality of care as inadequate was higher if the outcome of the case was bad. Additionally, GPs were also prone to outcome and hindsight bias.

To the best of our knowledge, no other study has evaluated implicit bias comparing patients with a functional disorder (fibromyalgia, CFS, IBS) and a somatic past medical history. Implicit bias was found among physicians and nurses for many patient characteristics, such as age/gender, race, socio-economic status of patients, mental illness, weight, and disability [20]. Many studies have shown differences in analgesia prescription for different races and sex, with lower analgesia prescriptions for black people [8–10], and women [21,22]. In our study, analgesia prescription was similar for men and women. An important difference with our study is that we did not show a picture of a men or women in the case, which was done in previous studies. Nonetheless, in our study men received less often an ultrasound to exclude appendicitis. Many participants believed that men have a lower risk for an appendicitis than women, but even after correction for this variable, implicit bias persisted with fewer ultrasounds performed in men. More importantly, patients with a functional disorder received fewer ultrasounds to exclude an appendicitis, received less opioids for chest pain, and troponins were less often performed if adjusted for other variables.

Years of clinical experience did not affect the presence of bias in our study which is similar to the study of Gupta *et al*. [5] However, a previous study from 1991 investigating framing bias found more presence of bias in residents than in experienced physicians [23]. We recognize that the relationship between clinical experience and cognitive bias is complex and influenced by various factors. For example, physicians may become better in recognizing potential bias and may be better in using heuristic thinking [24], but this pattern recognition may also lead to diagnostic overshadowing and overconfidence bias.

The judgment of the quality of care should be unaffected by the patient’s outcome, but it is likely that cases with a poor outcome will be investigated more often than with a good outcome, while care may have been similar. The quality of care can be approached in two ways. The safety-I logic believes that safety is the absence of failure, and safety and quality of care are best achieved by minimizing performance variation and maximizing compliance with clinical standards, guidelines, and regulations [7]. On the contrary, the safety-II perspective focusses on situations where safety is actually present in everyday work that goes well [7,25]. Central to Safety-II is that safety is a consequence of collective efforts to adapt to dynamic conditions and uncertainty. The main problem with Safety-I is that quality of care is investigated in case of an adverse event or incident, while outcome and hindsight bias are very hard to overcome. Furthermore, for cases in which the outcome of the patient was excellent, the quality of care provided could still have been poor, but this will be missed if only cases with a bad outcome are investigated. Therefore, more focus on a Safety-II-approach, which includes evaluation of all cases despite their outcome, may improve quality of care and improve individual well-being [7].

Our findings imply that professionals may need training to recognize implicit bias. As suggested, awareness is the first step to overcome implicit bias in medicine, and educational frameworks have been suggested for health profession educations [26]. Several methods could help to reduce the effect of implicit bias. First, Freund *et al*. suggested that cross-checking between EPs during shifts may reduce adverse events [27]. Cross-checking may also help to prevent the effects of implicit bias. Secondly, relying on clinical decision tools or flow diagrams may help to prevent disparity in ED treatment for vulnerable groups. Thirdly, teaching could be more focused on this theme by using the suggested educational frameworks and specific intervention which has shown to reduce implicit bias [26,28]. Nonetheless, it is well recognized, that in fast-paced environments such as the ED, it is hard to overcome cognitive bias. Only a few studies examined the relation between the ability to identify cognitive bias and a decrease in errors, which showed no benefit using debiasing strategies [29].

This study has several limitations. First, case vignettes are hypothetical and have a limited amount of supporting text, which differs from real case investigations. However, more information may feed hindsight bias as clinicians may search for the confirmation of a bad outcome such as atypical ECG changes, which may not have been specific during the actual ED visit. Therefore, outcome and hindsight bias may have been underestimated in our study. Secondly, we have not performed an Implicit Association Test (IAT) in participants to study implicit bias, which is considered the most convincing test in combination with measuring treatment in the actual world. We believe this could have strengthened our results, but would have made the study more complex. Thirdly, only one participant had experience with disciplinary law committees, and thus we could not study whether having this experience would reduce outcome bias. Lastly, the response rate was relatively low with underrepresentation of some subgroups. However, the total number of physicians included was well above the calculated sample size.

In summary, outcome, hindsight, and implicit bias are affecting clinical decision-making and retrospective judgment of clinical cases in the acute care.

## Acknowledgements

B.C., B.D.G., M.M., and W.D. devised and designed the study, contributed to the analyses, and edited the manuscript. P.P. and L.V. collected and analyzed data and wrote the manuscript. B.C. takes full responsibility for the study. All authors have read and approved the manuscript.

Ethics committee approval: The study was approved by the medical ethics committee of the Máxima Medical Center (N22.025).

### Conflicts of interest

There are no conflicts of interest.

## Supplementary Material


