# Supplementary material for: Using case vignettes to study the presence of outcome, hindsight, and implicit bias in acute unplanned medical care: a cross-sectional study
Source: Eur J Emerg Med. 2024 Feb 16;31(4):260–6. doi: 10.1097/MEJ.0000000000001127 (PMC11198948; doi:10.1097/MEJ.0000000000001127)
Supplement: Supplementary file 1 [file ejem-31-260-s001.pdf]

Supplemental digital content

Supplemental digital content 1: Six clinical vignettes

The four clinical scenarios for objective 1 were translated by two Dutch Emergency Physicians working in English speaking countries before they were used. Below, the English version of each scenario is presented. Participants received the vignette without an outcome, or with a positive or negative outcome.
Vignettes 5 and 6 were used for objective 2, to study implicit bias. These cases were presented to the participants in Dutch, but they are here presented in English.

Case 1
A 30-year-old woman presented to the Emergency Department with a severe, left-sided headache. The headache was of gradual onset during 2 hours. There was mild photophobia and nausea, but the patient did not vomit. Fifteen minutes before the headache developed, the patient saw a flickering scotoma in her visual field.

The patient had presented to an Emergency Department twice previously, with similar complaints. A neurologist diagnosed migraine.
The first presentation two years ago was nearly identical to the current episode, and a CT brain performed at the time was normal. At the second presentation, patient responded well to metoclopramide and morphine.
She relates that current symptoms are similar to those of previous visits. The patient had no other pertinent medical history. There is no history of drug and/or alcohol use, or high-risk sexual contact. Patient has not travelled recently and has never lived abroad. She has no other systemic complaints.

The documented physical examination included normal vital signs, normal fundi and vision, no increased ocular pressure or sinus tenderness, and a normal neurologic examination including cranial nerves, motor skills, sensibility, and coordination.
Patient is given 10 mg of metoclopramide and 10 mg of morphine intramuscularly. After an hour, the pain had decreased, with a pain score of 3 out of 10, and she wanted to go home to sleep. Neurological examination is unchanged.
Patient was discharged with paracetamol/codeine and was brought home by a friend.

Outcomes:
Good outcome: The patient was completely symptom-free at home after 1 day and had no residual symptoms.

Poor outcome: The patient was found dead at home 24 hours later. Autopsy revealed a ruptured arteriovenous malformation.

Case 2
A 46-year-old man came to the Emergency Department with abdominal pain, nausea, vomiting and diarrhea. The night before, the patient ate at a roadside restaurant, but none of his dining party were ill. Twelve hours after eating, the patient developed nausea. He vomited once and had diarrhea twice, with no blood or mucus admixture. Following this, he developed generalized abdominal pain.

His medical history included type 2 diabetes mellitus and hypercholesterolemia. Patient denied alcohol use, the social history did not provide additional details. There was no chest pain, shortness of breath or other systemic symptoms. He had not travelled recently, nor had he taken antibiotics.
On physical examination, the patient was subfebrile with a temperature of 37.9°C, pulse 95/min, blood pressure 115/89 mmHg and respiratory rate 19/min. There was mild pressure tenderness in the lower abdomen and lower right abdomen, without rebound, guarding or release pain. Urogenital examination was normal; a rectal examination was not performed. The patient received antiemetics and two litres of fluid o intravenous fluid. Laboratory tests showed leukocytes of 13x109/L. Other results, including lipase, metabolic profile, and urinalysis were normal.

Four hours later, the patient felt much better. He no longer had pain in the lower right abdomen and only minimal residual pain in the rest of the lower abdomen. His vital signs have normalized, the patient tolerated PO challenge and was discharged home with the advice to return if symptoms or concerns worsen.

Outcomes
Good outcome: On routine telephone follow-up, the patient stated he felt better and was scheduled for an annual check-up appointment with his primary care physician in two days. During that appointment, he endorsed complete resolution of his symptoms.

Poor outcome: The patient returned 2 days later with a temperature of 39.4°C and a rigid abdomen. Emergency laparotomy revealed a perforated appendix. Postoperatively, the patient had difficulty and was hospitalized for 4 weeks. Subsequently, he was readmitted twice for bowel obstruction.

Case 3

A 29-year-old otherwise healthy man was brought in by ambulance after a single-car motor vehicle crash at 80km/h while changing radio stations. He was a restrained driver, did not hit his head, did not lose consciousness, and was ambulatory at the scene. He complained of some left upper quadrant pain. The patient denied alcohol or drug use. Initial vital signs in the ambulance were temperature 37.7°C, with a pulse of 110/min, a blood pressure of 90/50mmHg and a respiratory rate of 20/min. The patient arrived at the ED with spinal precautions in place. The patient's complete primary and secondary survey results were negative, except for some mild left upper quadrant tenderness over the ribs, without any abdominal bruising or seat belt sign. The FAST examination result was negative. The patient was cleared from the backboard and cervical collar. Chest and pelvis radiograph results were unremarkable. Serial haemoglobin levels remained unchanged, at 9mmol/L and normal. A urine dip test result was negative for blood. The patient received some paracetamol and ibuprofen for analgesia.

He was observed for 2 hours and discharged. Before discharge, a repeated examination documented improved but continued mild left upper quadrant tenderness and unchanged vital signs. Adequate return precautions were noted on the discharge instructions.

Outcomes

Good outcome: The patient returned 1 day later to the ED, with continued left upper quadrant pain and an unchanged physical examination result. An abdominal CT was obtained and demonstrated a nondisplaced rib fracture, without evidence of other injuries. The patient was discharged with analgesia and recovered well.

Poor outcome: Eight hours later, the patient returned to the ED by ambulance. He was hypotensive and tachycardic. In response to a FAST scan demonstrating free fluid and a haemoglobin level of 5mmol/L, the patient received aggressive transfusion with packed RBCs and then received emergency laparotomy. The surgeons were unable to control bleeding from a ruptured spleen, and despite continued aggressive resuscitation the patient was pronounced dead on the table.

Case 4

A 65-year-old man presented to the ED with substernal burning chest pain of 30 minutes' duration that began while he was moving air conditioning equipment. He had an acid taste in his throat and was burping frequently. He was a smoker and had hypertension. He denied nausea or vomiting. When questioned about diaphoresis, he stated that he had been sweating all day "because it was so damn hot out." He described the pain as 8 of 10, with radiation to his left arm. He stated that he had experienced shortness of breath after moving the air conditioning unit, but this improved with rest. His vital signs were normal.

Physical examination result was normal. Specifically, the physician observed the absence of JVD or carotid bruits, normal heart sounds without S3/S4, normal lungs without rales, a soft abdomen without bruits, good pulses in all extremities with no edema, and a normal neurologic examination result. An ECG revealed normal sinus rhythm, with T-wave inversion and 0.5 mm of ST depression in the anterior leads. He was given 1 adult aspirin, a "gastrointestinal cocktail," and 3 sprays of sublingual nitroglycerin, which improved his pain to 1 of 10. Laboratory testing for CBC count, chemistry panel, and troponin I was conducted on his arrival, and results were normal. After 1 hour, the treating physician sent the patient home with a diagnosis of GERD and instructions for follow-up with his primary physician for an outpatient stress test.

Outcomes

Good outcome: The patient followed up with his primary care physician the next day. Outpatient treadmill result was normal and he subsequently recovered well.

Poor outcome: The patient returned 8 hours later in cardiac arrest as a result of a massive myocardial infarction and subsequently died.

Case 5
A 54-year-old **Man/Woman** weighing 65kg, presents with pain in the lower abdomen in the last 1.5 days. His primary care physician sent him to the Emergency Department given the pain was localized in the right lower quadrant and a CRP of 12 was measured. He had **No past medical history / fibromyalgia, depression, chronic fatigue syndrome.**

The pain is continuous and there is no pain upon transportation. The pain started acutely and radiates to the right groin. **He/She** has no fever, vomited once, and has a decreased appetite. The stool has not changed. There is no dysuria or haematuria. Paracetamol 1000mg every 6 hours has insufficient effect on the pain. The NRS is 6.
On examination, a temperature of 37.5°C and heart rate of 95/min are measured. Other vital signs are not abnormal. **He/She** sits slightly hunched over and leaning to the right on the examination table. Physical examination shows no other abnormalities.

Case 6
A 46-year-old **Man/Woman** weighing 104kg, presents with thoracic pain at the level of the sternum since this morning (5 hours ago). **He/She has a known history of pneumonia, appendicitis, and distal radius fracture / Depression, whiplash, fibromyalgia and IBS.**

He/She has had this pain several days in the past week during exertion, however, the symptoms have progressively increased since this morning.
He/She presents to the Emergency Department without a referral from **his/her** primary care physician. The pain started in the middle of the thorax and did not radiate. **He/She** took two paracetamol and 500mg naproxen with no effect. The NRS is 6.
Vital signs show normal heart rate and a tension of 175/95 mmHg. Other vital signs are not abnormal. **He/She** is panicking and hyperventilating as a result. During physical examination, you see a restless **man/woman**, hear normal heart sounds and he indicates increased pain on compression of the thorax.
Troponins are measured and show a T0 of 7, other blood results are not abnormal.

Supplemental digital content 2: randomisation sequence

Randomisation sequence for objective 1

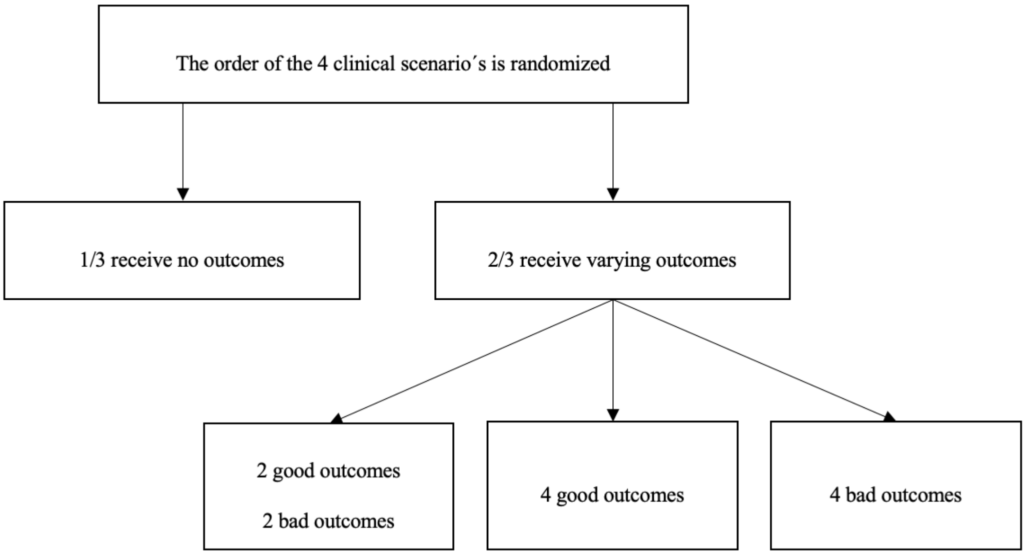

Randomisation sequence for objective 2

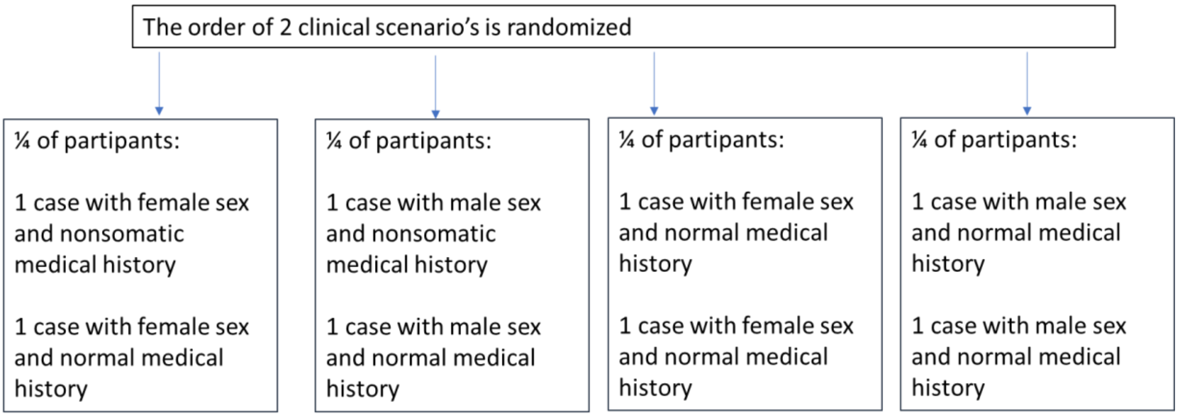

Supplemental digital content 3

Objective 1: Power 80%. $\alpha = 0.05$. We consider the answers on the 6-point likert scale (poor to outstanding) as a non-normally distributed ordinal outcome, as they can be skewed to left or right depending on the quality of the scenario. Because all vignettes may have a different mean/median on the likert scale, we estimated the mean as 'average (3)' with an SD of 0.5, based on the bar graphs in previous studies.(1-4) A change of one step on the likert scale is considered relevant (i.e. average to below average or average to good). Because of the skewed data, we first log transform the 3 and 2 points and the 3 and 4 points as given on the likert scale, and assume the log transformation is symmetric. The mean difference $\Delta = \ln(3) - \ln(2) = 1.099 - 0.69 = 0.41$, or $\ln(3)-\ln(4) = 0.29$. We chose 0.29 as Δ, because this would lead to a larger sample.

$N = (Z_{1-\beta} + Z_{1-\alpha/2})^2 (\sigma_1^2 + \sigma_2^2) / \Delta^2$

$Z_{1-\beta} = 0.84$, $Z_{1-\alpha/2} = 1.96$ (Table B1 of Altman 1991). $\Delta = 0.29$, $\sigma_1 = \sigma_2 = 0.5$

N=46 participants.

An estimate of the sample size for the Mann-Whitney test is calculated by division by 0.846,(5) yielding n = 54 per group. Because we compare three groups, we need to include 3x54 = 162 participants.

Objective 2: Power 80%. $\alpha = 0.05$. Outcome measures are dichotomous variables (yes/no) for both pain medication and diagnostics. We expect that 90% of the participants would prescribe opioids.(6) 20% difference between men and women or between different medical histories is considered relevant. We calculated we need therefore 73 participants in each group, in total 146 participants.

References

1. Banham-Hall E, Stevens S. Hindsight bias critically impacts on clinicians' assessment of care quality in retrospective case note review. Clin Med (Northfield Il). 2019;19(1):16.
2. Gupta M, Schriger DL, Tabas JA. The presence of outcome bias in emergency physician retrospective judgments of the quality of care. Ann Emerg Med. 2011;57(4):323-8. e9.
3. Caplan RA, Posner KL, Cheney FW. Effect of outcome on physician judgments of appropriateness of care. JAMA. 1991;265(15):1957-60.
4. Berlin L. Outcome bias. American Journal of Roentgenology. 2004;183(3):557-60.
5. de Winter JF, Dodou D. Five-point likert items: t test versus Mann-Whitney-Wilcoxon (Addendum added October 2012). Practical Assessment, Research, and Evaluation. 2010;15(1):11.
6. Weisse CS, Sorum PC, Sanders KN, Syat BL. Do gender and race affect decisions about pain management? J Gen Intern Med. 2001;16(4):211-7.

Supplemental digital content 4

Flow diagram for inclusion of participants.

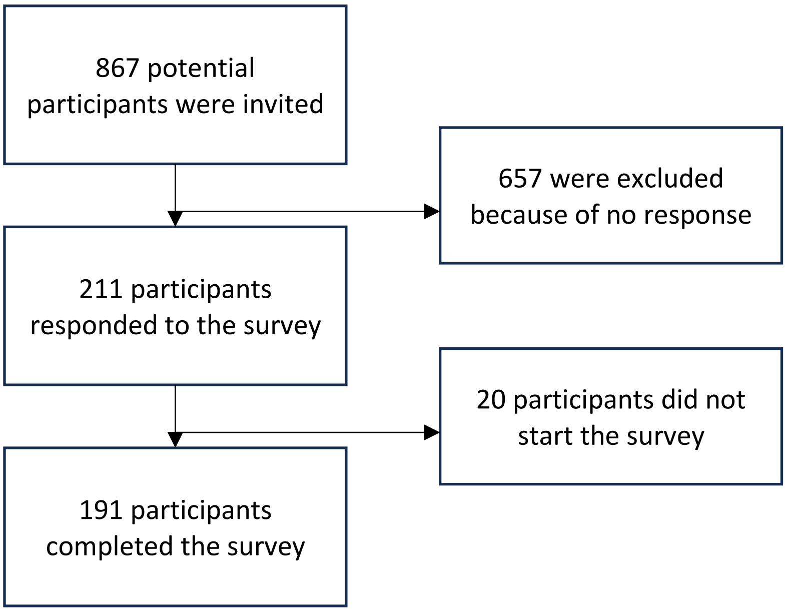

Supplemental digital content 5

The table below shows the results of the multivariable logistic regression analysis per case for objective 1. The odds ratios from the statistically significant variables are presented in bold.

Variables	Headache case	Abdominal pain case	Trauma case	Chest pain case
	AOR (95%CI)	AOR (95%CI)	AOR (95%CI)	AOR (95%CI)
Age	0.9 (0.8–1.1)	0.9 (0.8–1.1)	1.0 (0.9–1.1)	1.0 (0.8-1.2)
Male sex	2.9 (0.9–10.1)	1.5 (0.7–3.3)	0.7 (0.4–1.6)	0.5 (0.2–1.6)
Experience (years)	1.1 (0.9–1.2)	1.0 (0.9–1.2)	1.0 (0.9–1.2)	1.0 (0.8– 1.3)
EP in training	1.0	1.0	1.0	1.0
EP	1.6 (0.3–7.3)	1.0 (0.3–2.3)	1.4 (0.5–4.4)	0.4 (0.1–1.5)
GP	0.4 (0.1–2.4)	1.9 (0.4–9.7)	1.1(0.2–5.5)	1.1(0.2–7.6)
Other	0.2 (0.0–1.3)	1.3 (0.2–8.0)	2.1 (0.5–9.2)	/
Randomisation 1	1.0	1.0	1.0	1.0
Randomisation 2	0.5 (0.1-3.4)	1.2 (0.4-3.9)	2.1 (0.7-6.6)	2.3 (0.6-8.6)
Randomisation 3	1.3 (0.2-7.9)	3.3 (0.4-30.2)	0.4 (0.0-3.8)	5.0 (0.4-64.6)
Negative outcome	1.0	1.0	1.0	1.0
No outcome	0.4 (0.1–3.4)	**8.1 (3.1-21.1)**	**3.4 (1.2-10.2)**	4.5 (0.5-43.8)
Positive outcome	1.3 (0.2–8.0)	**9.9 (3.2-30.7)**	**4.9 (1.5-15.7)**	**12.0 (1.2-118.8)**

AOR: Adjusted Odds Ratio, 95% CI: 95percent confidence interval, EP: emergency physician, GP: General Practitioner

Supplemental digital content 6

Multivariable logistic regression analyses were performed to investigate whether other factors affected the prescription of pain relief, opioids, GTN, and additional investigations including ultrasound, electrocardiogram, and troponins for objective 2. The odds ratios from the statistically significant variables are presented in bold.

Variables	Abdominal pain case (AOR 95% CI)			Chest pain case (AOR 95% CI)				
	**Pain relief**	**Opioid**	**Ultrasound**	**Pain relief**	**Opioid**	**GTN**	**ECG**	**Troponins**
**Participant characteristics**								
Age	1.0 (0.8-1.3)	1.1 (1.0-1.2)	0.9 (0.8-1.1)	1.0 (0.9-1.1)	1.0 (0.9-1.1)	1.0 (0.8-1.1)	10. (0.8-1.3)	1.0 (0.9-1.2)
Male sex	0.5 (0.1-3.4)	0.8 (0.4-1.5)	0.7 (0.3-1.8)	0.7 (0.3-1.4)	0.5 (0.3-1.0)	0.8 (0.3-2.4)	0.0 (0.0-1.4)	1.0 (0.4-2.5)
Experience (years)	0.9 (0.7-1.2)	1.0 (0.9-1.1)	1.1 (0.9-1.3)	1.0 (0.9-1.1)	1.0 (0.9-1.2)	1.0 (0.9-1.2)	0.9 (0.7-1.1)	1.0 (0.9-1.2)
EP in training	1.0	1.0	1.0	1.0	1.0	1.0	1.0	1.0
EP	1.0	0.7 (0.3-1.9)	1.0 (0.2-4.5)	0.9 (0.3-2.9)	1.8 (0.6-5.0)	0.8 (0.1-4.6)	/	0.8 (0.2-3.7)
GP	1.0	0.4 (0.1-1.4)	0.6 (0.1-4.0)	0.5 (0.1-2.1)	0.3 (0.1-1.4)	0.3 (0.0-2.2)	0.5 (0.0-29)	0.3(0.1-1.6)
Other	1.0	0.4 (0.1-4.0)	1.2 (0.2-8.4)	0.2 (0.1-1.0)	0.8 (0.2-3.1)	0.5 (0.1-5.0)	1.2 (0.0-114)	01.9 (0.2-23)
**Case characteristics**								
Male case	1.4 (0.2-7.8)	0.9 (0.5-1.6)	**0.3 (0.1-0.8)**	0.8 (0.4-1.7)	0.7 (0.4-1.4)	0.6 (0.2-1.9)	0.3 (0.0-3.5)	0.4 (0.2-1.1)
Somatic case	2.8 (0.5-15.9)	1.8 (1.0-3.3)	**6.3 (2.1-18.8)**	1.2 (0.6-2.4)	**2.2 (1.1-4.3)**	2.1 (0.7-6.4)	3.8 (0.3-46)	**2.8 (1.1-7.4)**
**Explicit bias questions**								
PMH equal risk	1.0	1.0	1.0	1.0	1.0	1.0	1.0	1.0

Nonsomatic higher risk	/	0.7 (0.1-9.5)	/	0.9 (0.4-1.9)	1.3 (0.6-2.5)	1.1 (0.3-3.5)	0.7 (0.0-20)	0.9 (0.3-2.2)
Nonsomatic lower risk	/	3.2 (0.6-18.5)	1.0 (0.1-11.0)	/	/	0.2 (0.0-2.7)	**0.0 (0.0-0.7)**	0.5 (0.0-5.0)
Sex equal risk	1.0	1.0	1.0	1.0	1.0	1.0	1.0	1.0
Men higher risk	0.6 (0.1-3.4)	1.4 (0.7-2.6)	0.5 (0.2-1.3)	0.7 (0.3-1.6)	1.0 (0.4-2.1)	1.3 (0.4-4.1)	0.4 (0.0-54)	1.9 (0.7-5.9)
Men lower risk	0.2 (0.0-3.8)	0.6 (0.1-3.6)	**0.1 (0.0-0.4)**	1.0 (0.2-5.8)	0.8 (0.2-3.7)	1.1 (0.1-12)	/	1.6 (0.2-11)
**Outcome of vignette 2 or 4**								
No outcome	1.0	1.0	1.0	1.0	1.0	1.0	1.0	1.0
Negative	0.3 (0.0-2.9)	1.6 (0.7-3.3)	1.7 (0.6-5.2)	0.9 (0.4-2.1)	0.7 (0.3-1.5)	**4.6 (1.1-19.4)**	/	2.5 (0.7-8.4)
Positive	0.3 (0.0-2.8)	0.9 (0.4-2.0)	1.7 (0.6-5.3)	0.8 (0.3-2.0)	0.8 (0.4-1.7)	1.6 (0.5-5.2)	4.8 (0.1-220)	0.6 (0.2-1.7)

AOR: Adjusted Odds Ratio, 95% CI: 95percent confidence interval, EP: emergency physician, GP: General Practitioner

Explicit bias questions: Whether men or women and patients with a PMH of fibromyalgia, depression, and CFS or no PMH have an equal risk (similar, lower, higher) to have an appendicitis or an acute coronary syndrome.

The outcome of vignette 2 was added to the regression analysis for the abdominal pain case, and the outcome of vignette 4 for the chest pain case.
